# Simultaneous Presentation of Breast Cancer and Solitary Rib Plasmacytoma Mimicking Breast Cancer Metastasis: A Report of a Rare Case

**DOI:** 10.7759/cureus.87237

**Published:** 2025-07-03

**Authors:** Diana C Correa-Sandoval, Javier Gonzalez Reyes, Diego A Guajardo Nieto, Jose L Guzman Murguia

**Affiliations:** 1 Vice-Rectorate for Health Sciences, Universidad de Monterrey, San Pedro Garza Garcia, MEX; 2 Department of Breast Care, Hospital Angeles Valle Oriente, San Pedro Garza Garcia, MEX

**Keywords:** breast cancer, diagnostic challenge, dual malignancies, plasmacytoma, staging

## Abstract

In the realm of oncology, the detection of lytic bone lesions in patients with breast cancer commonly raises suspicions of metastatic disease, due to breast cancer's known propensity to metastasize to bone. However, the clinical journey of diagnosing such lesions demands a discerning differential diagnosis, particularly when encountering rare entities like solitary rib plasmacytoma alongside breast cancer. This case report elucidates the diagnostic challenges and clinical implications of managing a patient with dual malignancies, emphasizing the importance of an exhaustive and nuanced diagnostic approach. It highlights the need to differentiate between metastatic breast cancer and other primary malignancies, such as plasmacytoma, which can critically influence staging, prognosis, and therapeutic strategies. This case report presents a 64-year-old female patient with a diagnosis of breast cancer who was found to have a co-existing rib plasmacytoma and highlights the diagnostic challenges posed by dual malignancies. This patient presented with a single lytic bone lesion in the rib at the time of breast cancer diagnosis and staging workup. Despite initial suspicion of metastatic breast disease, the atypical presentation warranted further investigation. The diagnostic workup revealed a solitary rib plasmacytoma, emphasizing the importance of a comprehensive diagnostic approach. Following confirmation of non-metastatic plasmacytoma, the staging of breast cancer was revised. This case illustrates how breast cancer, being the most common global malignancy, can simultaneously present with other malignancies that can imitate a different cancer staging.

## Introduction

Breast cancer, a leading malignancy in women worldwide, manifests in diverse forms and histologies. The detection of a lytic bone lesion in a patient with breast cancer typically raises the suspicion of metastatic disease, given the propensity of breast cancer to spread to bone. However, the clinical significance of identifying such lesions extends beyond the mere presence of metastasis; it necessitates a rigorous differential diagnosis to distinguish between metastatic breast cancer and other primary bone pathologies that can mimic metastasis. The latter, especially when located in the rib, is an exceedingly rare and distinct entity from breast malignancy, often leading to a pivotal shift in staging, prognosis, and therapeutic strategies [1]. This case describes the presentation of a solitary rib plasmacytoma that was incidentally identified during the imaging workup as part of the breast cancer evaluation. It underlines the importance of searching for other diagnostic entities when presented with an unusual bone lesion location in a breast cancer patient. The simultaneous presentation of breast cancer with a solitary rib plasmacytoma represents an extraordinary diagnostic anomaly, previously unreported in the literature. This report documents a unique case of a patient with this rare combination, emphasizing the rarity and diagnostic complexities involved.

## Case presentation

A 64-year-old woman visited our clinic, asymptomatic, to request a second opinion with a diagnosis of left breast cancer. She had undergone a partial mastectomy followed by sentinel lymph node dissection at a different institution nine days earlier. The patient presents with a strong family history of breast cancer, including her sister and a first cousin who were both diagnosed at 45. She had a past abdominal hysterectomy for uterine myomatosis. Physical examination revealed a surgical scar on the left breast and axilla without complications. 

The pathology review reported grade two infiltrating ductal carcinoma, moderately differentiated, associated with ductal carcinoma in situ, including lymphatic and perineural invasion, with one sentinel node showing macrometastasis and capsular invasion. Immunohistochemical analysis revealed estrogen receptor (ER) and progesterone receptor (PR) positivity, low Ki67 expression (8%), and HER2NEU negativity.

**Figure 1:**
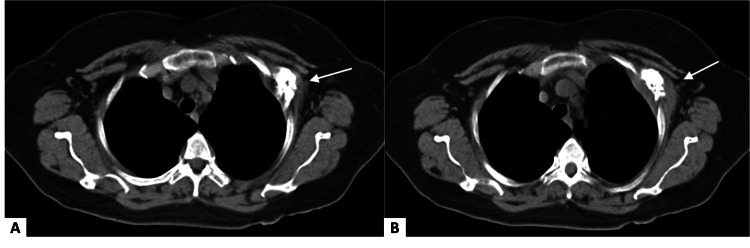
Chest CT scan Axial CT slices show extensive destruction involving the anterolateral portion of the left fourth costal arch, associated with a soft tissue mass (A, B).

A CT scan was performed to properly establish the extent of the breast carcinoma and determine its stage, revealing a lytic lesion in the 4th left costal arch (Figure 1). The patient exhibited no symptoms related to the rib lesion. This finding, initially suspicious for metastatic breast disease, led to further investigation. 

**Figure 2:**
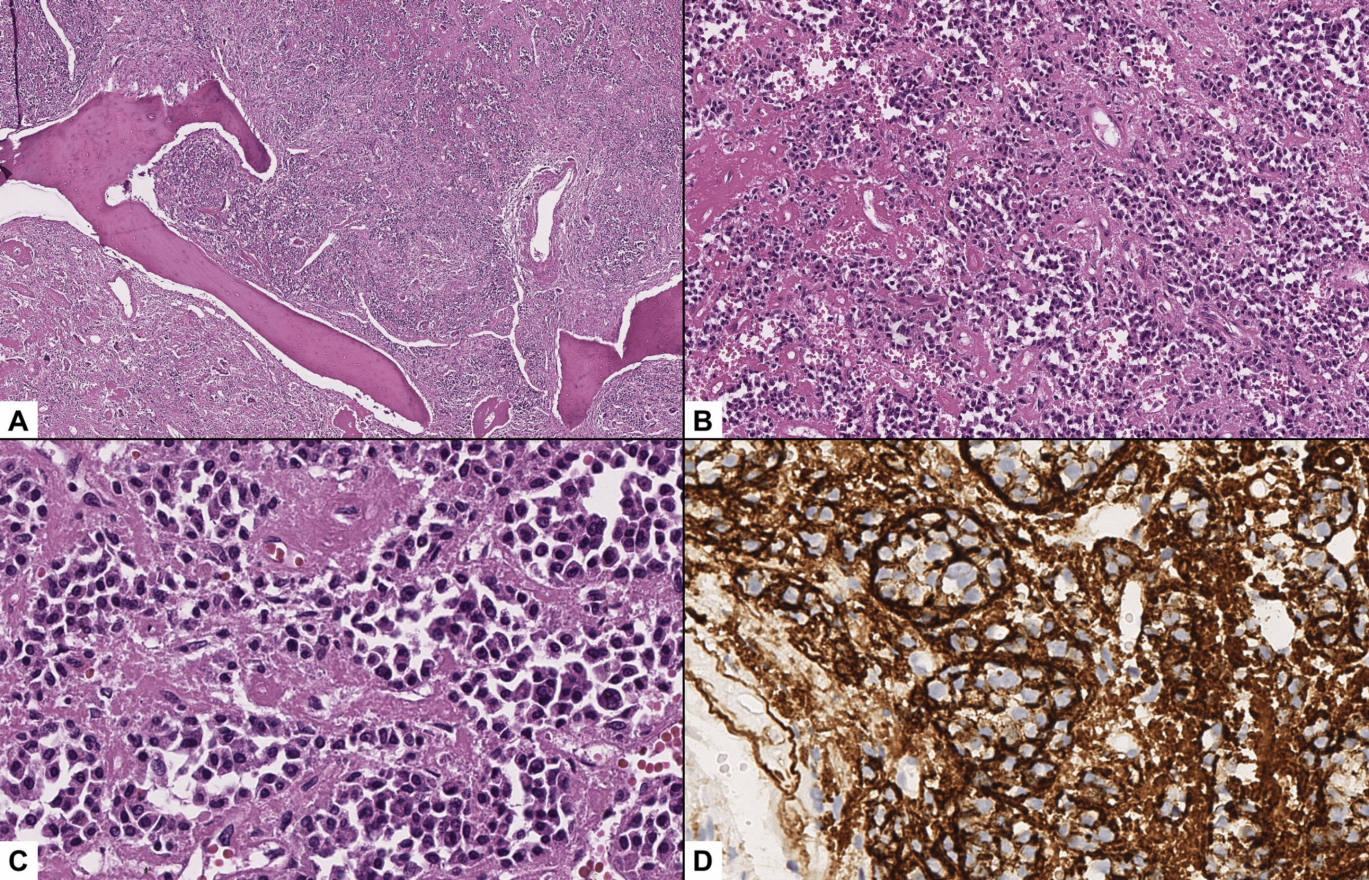
Plasmacytoma histopathological findings (A, B) The biopsy shows involvement of the bone tissue by a neoplasm composed of a monotonous population of cells arranged in clusters of various sizes, completely filling the intertrabecular spaces, and is accompanied by foci of hemorrhage (H&E, 40x, 100x). (C) The neoplasm is composed of clusters of mature plasma cells with abundant basophilic cytoplasm, perinuclear clearing referred to as perinuclear hof, an eccentric ovoid nucleus, and irregular chromatin (H&E, 400x). (D) Immunohistochemical staining for CD138 was diffusely positive in the neoplastic cells (400x).

To complete the standard treatment of that time, a radical axillary dissection was performed due to sentinel lymph node involvement identified on pathological examination. During the same procedure, a rib biopsy was obtained, revealing a proliferation of immature plasma cells. Immunohistochemistry was positive for CD138 and a Ki-67 proliferation index exceeding 20%, confirming a diagnosis of a plasma cell neoplasm consistent with plasmacytoma (Figure 2). 

A whole-body MRI was performed to assess the extent of the plasmacytoma and exclude the presence of additional lesions suggestive of multiple myeloma. The scan showed no further lytic lesions, no evidence of target organ damage, and no bone marrow involvement beyond the initially identified lesion. The diagnosis of solitary plasmacytoma of the rib was confirmed.

Following the diagnosis, the patient received adjuvant chemotherapy, radiotherapy, and tamoxifen for breast cancer and was referred for management by hematology for the plasmacytoma, undergoing 25 sessions of radiotherapy at 50 Gy, including the same field as the breast. Currently, the patient has remained disease-free for more than five years post treatment and continues to have annual follow-ups, showing no signs of recurrence for breast cancer and the plasmacytoma.

## Discussion

This case report unveils the uncommon simultaneous presentation of breast cancer and solitary rib plasmacytoma in a 64-year-old female patient, a scenario posing significant diagnostic and therapeutic challenges. The initial finding of an osteolytic rib lesion in a breast cancer patient typically triggers an evaluation for metastatic disease, a common progression of this malignancy [1]. However, the eventual diagnosis of solitary rib plasmacytoma, given its rarity and clinical presentation, underscores the importance of a comprehensive diagnostic approach.

Osteolytic lesions encompass the majority of bone metastasis in breast cancer patients, and these are analogous to the lesions in bone as solitary plasmacytoma develops. The most common initial site of metastasis in breast cancer is in the sternum and pelvis, prompting our attention to the possibility that the osteolytic lesion in the rib may be something distinct from metastasis [2]. The ribs are the most involved bones in a patient with multiple bone metastases, but an initial osteolytic lesion in the rib is only present in 17.5% of breast cancer patients [2]. Patients with extraskeletal cancer and solitary rib hot spots have been found in 88.2% of cases to have benign lesions [3]. While a scintigram is a different diagnostic tool than the CT scan, which identifies the osteolytic lesion in the left costal arch of this patient, the study displays that a significant number of cancer patients with bone lesions are later excluded from being metastatic [4]. 

The differential diagnosis for a rib lesion in this context is broad, encompassing metastasis from carcinoma, benign bone lesions, primary bone malignancies, multiple myeloma, and osteomyelitis, among others. After performing the biopsy, MRI was crucial not only for confirming the plasmacytoma via pathology and immunohistochemistry but also for verifying the lesion's singularity. This distinction is vital for differentiating solitary plasmacytoma from multiple myeloma, and it significantly impacts management strategies and prognosis [5, 6]. A solitary plasmacytoma of the bone is exceptionally rare, with an incidence of three per 1,000,000 annually, and accounts for only 4% of all cancerous plasma cell tumors [5]. This case highlights the essential role of thorough investigations in distinguishing between the mentioned possibilities, ensuring that treatment decisions are based on an accurate understanding of the patient's overall disease burden.

The conventional treatment for solitary rib plasmacytoma involves localized radiotherapy, administering doses of 40-50 Gy to achieve local control and minimize the risk of progression to multiple myeloma. This necessitates vigilant, long-term follow-up with regular imaging and serum monoclonal protein level assessments to detect any signs of disease progression or recurrence [7].

## Conclusions

The uniqueness of this case, undocumented in existing literature, demands tailored care and vigilant follow-up, involving specialists like breast surgeons and hematologists. This contribution highlights the critical role of collaborative efforts in managing rare oncological challenges. Finally, it demonstrates how a rare hematologic cancer can mimic an advanced stage of the most common neoplastic entity in women, breast cancer. By presenting the findings from this case, we aim to enrich the existing body of medical knowledge on rare cancer presentations. 
